# miR-592/WSB1/HIF-1α axis inhibits glycolytic metabolism to decrease hepatocellular carcinoma growth

**DOI:** 10.18632/oncotarget.9135

**Published:** 2016-05-02

**Authors:** Yan-Yan Jia, Jin-Yi Zhao, Bing-Ling Li, Kai Gao, Ying Song, Mei-You Liu, Xiao-Juan Yang, Yan Xue, Ai-Dong Wen, Lei Shi

**Affiliations:** ^1^ Department of Pharmacy, General Hospital of Guangzhou Military Command of People's Liberation Army, Guangzhou, Guangdong, P. R. China; ^2^ Department of Pharmacy, Xijing Hospital, Fourth Military Medical University, Xi'an, Shaanxi, P. R. China; ^3^ Department of Oncology, Xijing Hospital, Fourth Military Medical University, Xi'an, Shaanxi, P. R. China

**Keywords:** hepatocellular carcinoma, miR-592, hypoxia inducible factor-1α, glycolysis, WSB1

## Abstract

Hepatocellular carcinoma (HCC) cells rapidly switch their energy source from oxidative phosphorylation to glycolytic metabolism in order to efficiently proliferate. However, the molecular mechanisms responsible for this switch remain unclear. In this study, we found that miR-592 was frequently downregulated in human HCC tissues and cell lines, and its downregulation was closely correlated with aggressive clinicopathological features and poor prognosis of HCC patients. Overexpression of miR-592 inhibited aerobic glycolysis and proliferation in HCC cells *in vitro*. Conversely, knockdown of miR-592 promoted HCC growth in both subcutaneous injection and orthotopic liver tumor implantation models *in vivo*. Mechanistically, miR-592 downregulation in human HCCs was correlated with an upregulation of WD repeat and SOCS box containing 1 (WSB1). We further showed that miR-592 directly binds to the *3′-UTR* of the WSB1 gene, thus disrupting hypoxia inducible factor-1α (HIF-1α) protein stabilization. In turn, overexpression of WSB1 in HCC cells rescued decreased HIF-1α expression, glucose uptake, and HCC growth induced by miR-592. Collectively, our clinical data and functional studies suggest that miR-592 is a new robust inhibitor of the Warburg effect and a promising therapeutic target for HCC treatment.

## INTRODUCTION

Hepatocellular carcinoma (HCC) is the most common types of liver cancer and the third leading cause of cancer-associated death [1]. The difficulties of curing HCC are mainly due to an unclear elucidation of the heterogeneous genetic and epigenetic changes of HCC [2]. When HCC is highly proliferative, an increase in glycolytic flux is necessary to support its rapid requirement for nutrients [3]. However, therapeutics against HCC metabolism is a relatively unexplored area, which should deserve more attention.

microRNAs (miRNAs) are endogenous, small non-coding RNA molecules with a length of 18–25 nucleotides [4]. Its deregulation has been widely reported to be involved in the development of various cancers [5]. In recent years, miRNA has emerged as an important class of gene regulator in HCC development [6], and hopefully the study of its action mechanisms might provide new therapeutic intervention.

HIF-1α-induced transcriptional regulation controls several key genes crucial to deregulated glucose metabolism in cancers, that is, the Warburg effect [7]. However, it remains poorly understood about the role of miRNAs in HIF-1α-altered tumor cell metabolism. WD repeat and SOCS box containing 1 (WSB1) was recently identified as a new member of the suppressor of cytokine signaling (SOCS) box protein family, and its expression was upregulated in multiple types of human cancers [8]. More recent evidence suggested that WSB1 is a direct target of HIF-1α in a human HCC model [9]. Interestingly, WSB1 can in turn stabilize HIF-1α by protecting it from VHL-induced degradation [10]. Thus it is possible that WSB1 may promote HCC progression through altering cancer cell metabolism. However, very little was known about it.

In this study, for the first time, we investigated the aberrant status of miR-592 and its potential target gene to explore this miRNA's cancer-associated functions in HCC. Our results indicate that miR-592 is a robust inhibitor of the Warburg effect by targeting *WSB1*/HIF-1a axis, and more importantly, the newly identified miR-592 and its downstream pathway might serve as potential therapeutic targets and novel prognosis indicators in HCC.

## RESULTS

### miR-592 is frequently downregulated in human HCC tissues and cell lines, which associates with low postoperative survival rate

**Figure 1:**
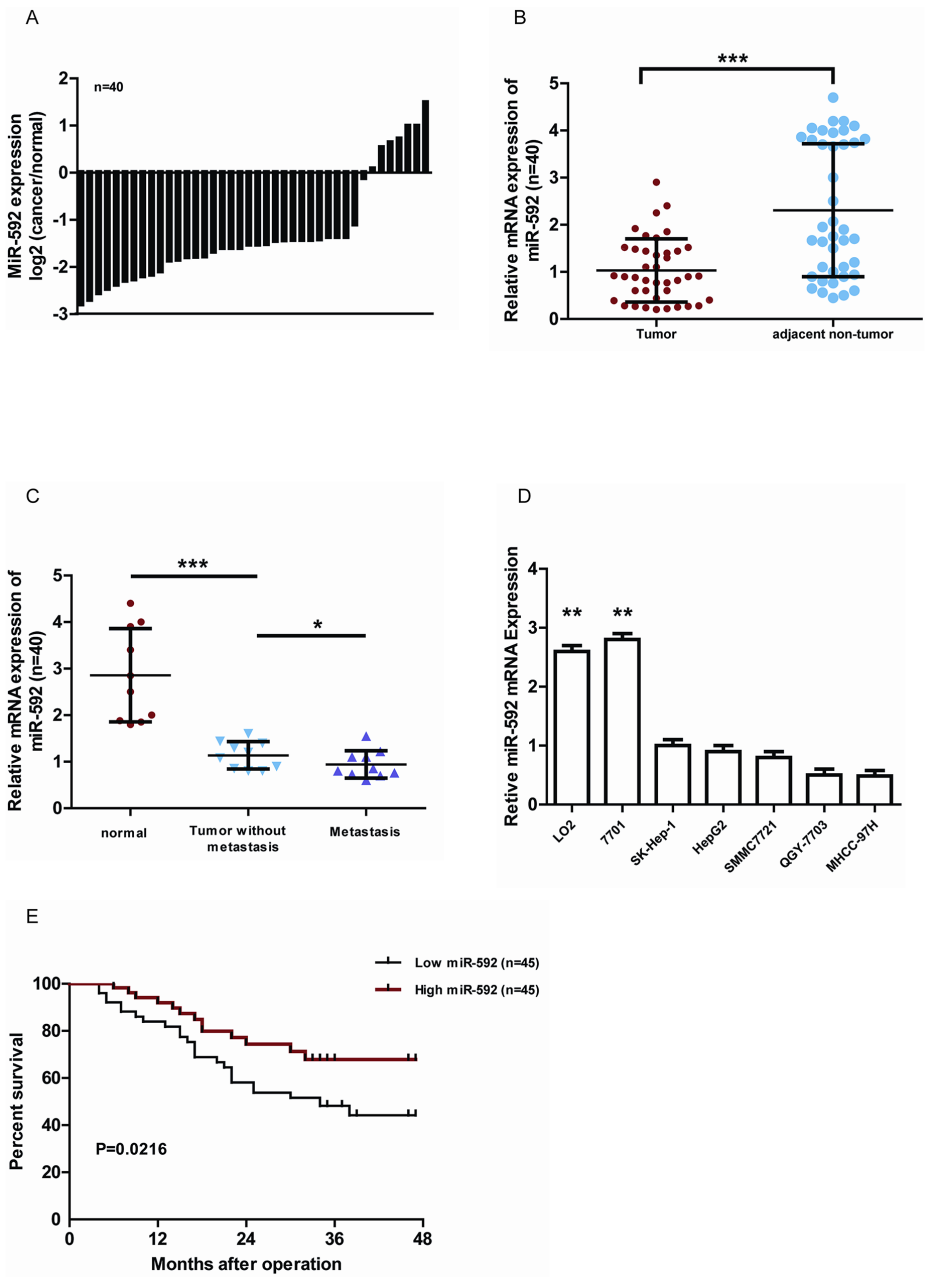
MiR-592 is frequently down-regulated in human HCC and associates with poor clinicopathologic features **A.** qRT-PCR analysis of miR-592 expression in 40 pairs HCC tissues and their adjacent normal tissues. **B.** The expression of miR-592 in HCC tissues was significantly lower than in corresponding normal tissues. **C.** The expression of miR-592 in HCC tissues, tissues with lymph node metastases, and their adjacent normal tissues, n=10/group. **D.** qRT-PCR analysis was used to investigate the expression levels of miR-592 in five HCC cell lines (HepG2, SK-Hep1, MHCC-97H, SMMC7721 and QGY-7703) compared with two nonneoplastic liver cell lines (LO2 and 7701). **E.** miR-592 expression was inversely associated with the overall survival of 90 HCC patients. The median was used as the cut-off value to divide patients into low and high expression groups. The mean and SD of three independent experiments performed in triplicate are shown.

To determine the expression of miR-592 in HCC, we analyzed 40 cases of HCC tissues and adjacent non-tumorous liver tissues using quantitative real-time PCR. Compared with the control tissues, the expression of miR-592 was significantly decreased in 33 of 40 HCC tissues (Figure 1A). And the median level of miR-592 in HCC tissues was obviously lower than in corresponding normal tissues (Figure 1B). Moreover, compared with primary HCC tissues and the adjacent normal tissues, tissues from lymph node metastases expressed lower levels of miR-592 (Figure 1C). Consistent with these clinical findings, the expression of miR-592 was significantly decreased in five HCC cell lines compared with two normal liver cell lines (LO2 and 7701) (Figure 1D). In addition, we analyzed the correlationship between miR-592 expression and the patients' prognoses. According to the median level of miR-592 expression, the patients (90 cases) were divided into two groups as either high expression (45 patients) or low expression (45 patients). It was found that the overall survival of HCC patients with low miR-592 expression was shorter than that of patients with high miR-592 expression (Figure 1E) Together, our data indicate that downregulation of miR-592 expression closely associates with poor HCC prognosis, suggesting that miR-592 participates in HCC progression.

### MiR-592 inhibits HCC cell proliferation and tumor growth

**Figure 2:**
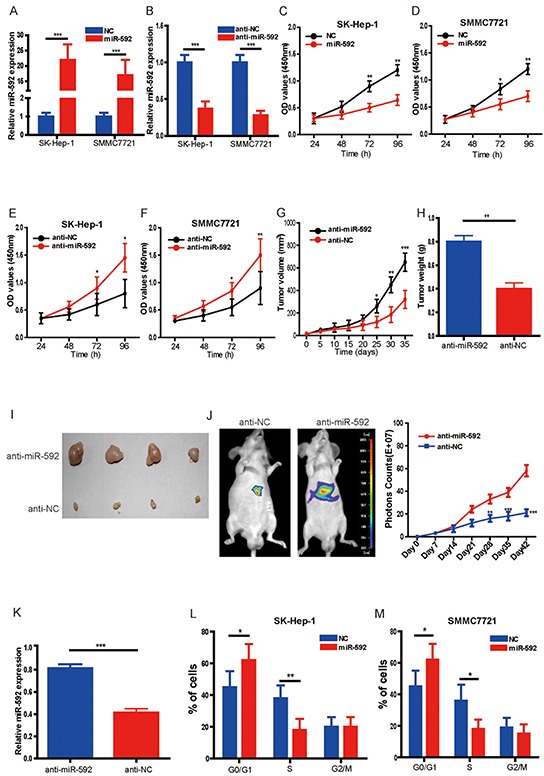
MiR-592 inhibits HCC cell proliferation and tumor growth **A.** and **B.** SK-Hep-1 and SMMC772 cells were transfected with hsa-miR-592 mimics, anti-miR-592 or negative control, respectively. The expression of miR-592 was confirmed by qRT-PCR. **C.** and **D.** Overexpression of miR-592 significantly decreased cell proliferation of SK-Hep-1 and SMMC772 cells compared to the negative controls using CCK-8 assay. **E.** and **F.** Knockdown of miR-592 markedly enhanced cell proliferation compared to the negative controls using CCK-8 assay. SK-Hep-1 cells transfected with anti-miR-592 or negative control (anti-NC) were subcutaneously injected into the flank region of nude mice. **G.** Tumor volumes and **H.** the tumor weights were measured; **I.** representative images of tumor were shown. **J.** Representative bioluminescence images corresponding to SK-Hep-1 orthotopic hepatic tumors (left panel). Volume of SK-Hep-1 orthotopic tumours was determined at different time points. **K.** The expression of miR-592 in tumor tissues was analyzed by qRT-PCR. **L.** and **M.** Cell cycle distribution of SK-Hep-1 and SMMC772 cells transfected with miR-592 mimics or NC was examined using flow cytometry. The mean and SD of three independent experiments performed in triplicate are shown.

To investigate the biological function of miR-592 in HCC, SK-Hep-1 and SMMC7721 cells were transfected with miR-592 mimic or control (NC), or miR-592 was also knocked down by transiently transfecting antisense oligonucleotides (anti-miR-592 or anti-NC) in the same cell lines. qRT-PCR was used to confirm transfection efficiency (Figure 2A and Figure 2B). Compared with the negative controls, overexpression of miR-592 in SK-Hep-1 and SMMC7721 cells significantly decreased cell number (Figure 2C and Figure 2D) whereas knockdown of the endogenous miR-592 markedly increased the viability of these cells (Figure 2E and Figure 2F).

To confirm this role of miR-592 applied *in vivo*, we tested whether miR-592 knockdown would promote tumour growth in SK-Hep-1 orthotopic tumour models and mouse subcutaneous xenograft models. Firstly, SK-Hep-1 cells were transfected with agomiR-592 or negative control (agomiR-NC), and then subcutaneously injected into the flank region of nude mice. It was found that compared to the agomiR-NC group, the tumor volumes, sizes and weights in agomiR-592 group were markedly increased (Figure 2G, Figure 2H and Figure 2I). Secondly, our in vivo orthotopic tumour study also further reinforced our in vitro results, and established the potential significance of miR-592 in HCC progression (Figure 2J). qRT-PCR analysis of tumor tissues confirmed that the expression of miR-592 was significantly decreased (Figure 2K). In order to validate the therapeutic value of miR-592, tumor cells with miR-592 overexpression were injected into mice to examine the inhibition of tumor growth. As shown in supplementary Figure 1A, 1B, 1C, 1D and 1E, miR-592 overexpression significantly inhibited HCC growth in mice models. We also used flow cytometry to examine the cell cycle distribution of HCC cells. As shown in Figure 2L and Figure 2M, compared to cells transfected with negative controls, there was a significant increase in G0/G1 phase cells and decrease in S phase cells in miR-592 mimics-transfected SK-Hep-1 and SMMC772 cells, whereas miR-592 knockdown cells displayed the opposite data (data not shown). In addition, as shown in supplementary Figure 1F, miR-592 inhibits the migration of HCC cells in vitro. Collectively, our findings suggest that miR-592 decreases HCC cell proliferation *in vitro* and tumorigenicity *in vivo*.

### miR-592 directly targets WSB1 in HCC cells

**Figure 3:**
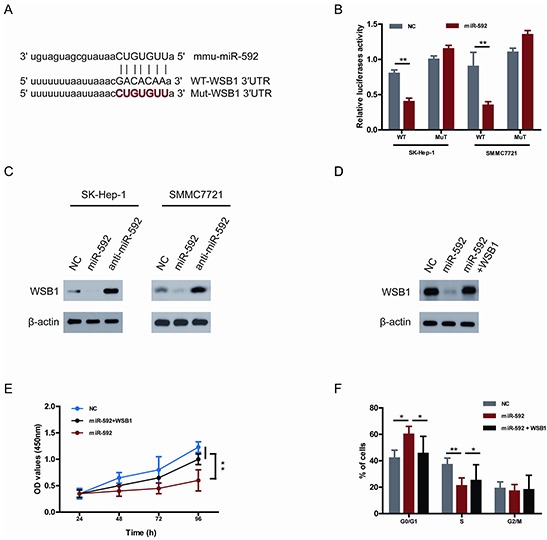
WSB1 is a bona fide target gene of miR-592 and functional downstream of miR-592 in HCC cells **A.** Sequence alignment of wild-type (WT) and mutated (Mut) putative miR-592-binding sites in the 3′-UTR of WSB1. **B.** Relative luciferase activities of plasmids carrying WT or mut WSB1 3′-UTR in SK-Hep-1 and SMMC7721 cells cotransfected with miR-592 mimics or NC. **C.** The expression levels of WSB1 in SK-Hep-1 and SMMC7721 cells were analyzed using western blot. **D.** SK-Hep-1 cells were co-transfected with miR-592 mimics or NC and WSB1 plasmid without 3′-UTR, the expression levels of WSB1 were analyzed using western blot. The effect of miR-592 and WSB1 on **E.** HCC cell proliferation and **F.** cell cycle progression. The mean and SD of three independent experiments performed in triplicate are shown.

To uncover the underlying mechanism accounting for miR-592-induced inhibition of HCC cells proliferation, bioinformatics research was performed to predict miR-592 targets using PITA and miRanda [11]. Given that WSB1 increases one of the most important glycolytic proteins-HIF-1α and contributes to human malignancy [10], thus WSB1 was predicted as a potentially functional target gene of miR-592. As shown in Figure 3A, one predicted binding site in the WSB1 3′-UTR with a perfect complementarity to the seed region of the miR-592 was identified. To demonstrate whether the 3′-UTR of WSB1 could be a genuine target of miR-592, a dual-luciferase reporter system was constructed. WSB1 3′-UTR sequences containing wild type or mutated binding site of miR-592 were cloned into the psiCHECK2 vector, respectively, and co-transfected with the miR-592 mimics or NC into HCC cells. It was found that overexpression of miR-592 remarkably suppressed the luciferase activity of the reporter gene with the wild-type construct, but not with the mutant WSB1 3′-UTR construct in both SK-Hep-1 and SMMC7721 cells (Figure 3B), suggesting that miR-592 directly targeted the WSB1 3′-UTR. Moreover, overexpression of miR-592 also repressed the protein levels of WSB1 in both SK-Hep-1 and SMMC7721 cells. Conversely, suppression of miR-592 enhanced the expression of WSB1 in both SK-Hep-1 and SMMC7721 cells (Figure 3C).

To investigate whether the tumor suppressor role of miR-592 on HCC cell proliferation is mediated by inhibiting the expression of WSB1, SK-Hep-1 cells were co-transfected with miR-592 mimics and WSB1 plasmid without 3′-UTR. As shown in Figure 3D, miR-592-induced WSB1 downregulation was rescued following co-transfection. Moreover, the inhibitory role of miR-592 in proliferation and cell cycle progression was reverted under the condition of mutant WSB1 overexpression (Figure 3E and Figure 3F). Together, these results suggest that WSB1 is a bona fide target gene of miR-592 and functional downstream of miR-592 in HCC cells.

### The miR-592/WSB1 signal pathway inhibits glycolysis in HCC cells

**Figure 4:**
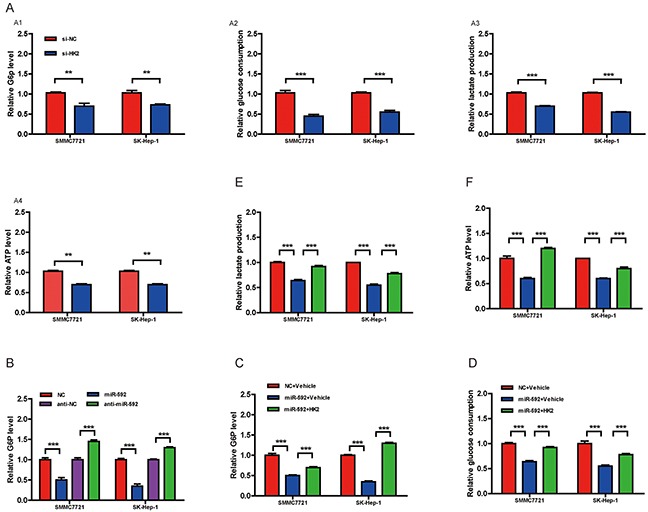
The miR-592/WSB1 axis inhibits glycolysis in HCC cells **A.** Measurement of the cellular G6P level, glucose consumption, lactate production, and cellular ATP level in HCC cells with knockdown of WSB1 using siRNA or anti-NC as control. **B.** Measurement of the cellular G6P level in HCC cells with miR-592 overexpression or downregulation. Abbreviation: NC, negative control. Measurement of the cellular G6P level **C.** glucose consumption **D.** lactate production **E.** and cellular ATP level **F.** in HCC cells with overexpression of WSB1. The mean and SD of three independent experiments performed in triplicate are shown.

Given that WSB1 promotes VHL ubiquitination and proteasomal degradation, thereby stabilizing HIF under both normoxic and hypoxic conditions, and HIF-1α has been shown to be a key transcription factor promoting glycolytic energy metabolism in cancer cells [10], we wondered if this newly discovered miR-592/WSB1 axis might inhibit glycolysis in HCC cells. As shown in Figure 4A, knockdown of WSB1 significantly decreased the cellular G6P level, glucose consumption, lactate production, and cellular ATP level in HCC cells. Consistently, overexpression of miR-592 greatly reduced the cellular G6P level in HCC cells, whereas knockdown of the endogenous miR-592 increased the G6P level (Figure 4B). Importantly, reintroduction of WSB1 reverted the miR-592-induced decrease in the cellular G6P level in HCC cells (Figure 4C). Overexpression of WSB1 protein in HCC cells also significantly abolished the suppression of miR-592 on glucose consumption (Figure 4D), lactate production (Figure 4E), and ATP level (Figure 4F), suggesting that miR-592 may suppress glycolysis by directly decreasing WSB1 expression in HCC cells.

### Knockdown of miR-592 enhances glycolysis by increasing WSB1-induced HIF-1α stability

**Figure 5:**
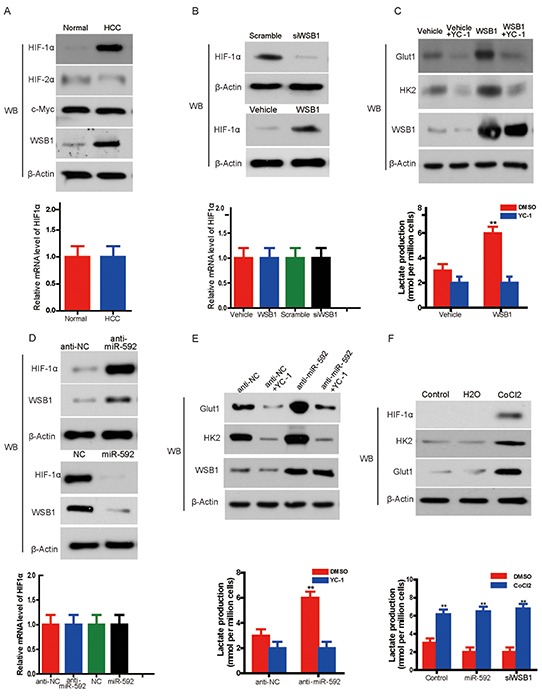
Knockdown of miR-592 enhances glycolysis by increasing WSB1-induced HIF-1α stability **A.** Western blot shows expression of HIF-1α, HIF-2α, c-Myc in HCC tissue and normal control (left panel). Quantitative RT-PCR shows the expression levels of HIF-1α mRNA in HCC tissue and normal control (right panel). **B.** The protein level of HIF-1α in SK-Hep-1 cells with overexpression or knockdown of WSB1 (left panel); mRNA level of HIF-1α in HCC cells with overexpression or knockdown of WSB1 (right panel). **C.** Expression of glycolysis-associated proteins (HK2 and Glut1) in HCC cells treated with the HIF-1α inhibitor YC-1 or overexpression of WSB1 (left panel); lactate production in the same cells (right panel). **D.** The protein level (left panel) or mRNA level (right panel) of HIF-1α with overexpression or knockdown of miR-592. **E.** The expression of HK2 and Glut1 in SK-Hep-1 cells treated with YC-1 and/or miR-592 knockdown (left panel); lactate production in HCC (right panel). **F.** The effect of CoCl2 on the expression of HK2 and Glut1 (left panel), and the effects of CoCl2 on lactate production (right panel) in SK-Hep-1 cells with WSB1 knockdown or miR-592 overexpression. The mean and SD of three independent experiments performed in triplicate are shown.

Because WSB1 can increase the stability of HIF-1α protein [10] and HIF-1α is one of key transcription factors in enhancing glycolysis [12], we hypothesized that HIF-1α may dictate the miR-592 knockdown-induced glycolysis in HCC cells. To confirm this idea, firstly, the expression of HIF-1α was examined. It was found that the expression of HIF-1α protein was markedly increased in HCC tissues, without significant changes for HIF-2α, or c-myc, or the levels of HIF-1α mRNA in HCC and normal control (Figure 5A), suggesting that HIF-1α accumulation in HCC is likely due to enhanced protein stability rather than increased transcription. Consistently, WSB1 overexpression enhanced HIF-1α protein level, whereas its knockdown decreased HIF-1α protein level, without changes for HIF-1α mRNA levels by both treatments (Figure 5B). To further confirm that miR-592 decreases HIF-1α stability via WSB1, the protein degradation of endogenous HIF-1α was measured in HCC cells with overexpression and knockdown of miR-592 and WSB1. As shown in supplementary Figure 2A and 2B, miR-592 significantly decreases HIF-1α stability which was reverted by WSB1 overexpression.

To determine whether HIF-1α is responsible for WSB1-enhanced glycolysis, we treated HCC cells with the HIF-1α inhibitor YC-1 [13]. WSB1 overexpression enhanced the expression of HK2 and Glut1 and lactate production in HCC cells, whereas YC-1 treatment reverted these increases (Figure 5C). In addition, miR-592 knockdown increased, whereas miR-592 overexpression decreased HIF-1α protein expression, but not mRNA level (Figure 5D). Knockdown of miR-592 also increased the expression of Glut1 and HK2 and lactate production; however, YC-1 rescued Glut1 and HK2 expression and lactate production enhanced by miR-592 knockdown (Figure 5E).

Consistent with these data, CoCl_2,_ a chemical inducer of HIF-1α protein [14], enhanced the expression of HK2 and Glut1 and lactate production in HCC cells (Figure 5F, upper panel). Although it was observed thatthe decreasing of lactate production upon overexpression of miR-592 or knockdown of WSB1 statistically significant compared to control in DMSO treated group, neither miR-592 overexpression nor WSB1 knockdown blocked the CoCl_2_ or HIF-1α-induced increase of HK2 and Glut1 expression and lactate production (Figure 5F, lower panel). Therefore, although miR-592 overexpression or WSB knockdown increased pVHL stability, CoCl_2_ inhibited PHD activity and disrupted the interaction between PHD and pVHL, thus miR-592 overexpression or WSB knockdown could not block the CoCls-induced lactate production. Together, our findings indicate that HIF-1α is responsible for the changes in glycolysis induced by miR-592 downregulation or WSB1 upregulation.

### Clinical significance of miR-592, WSB1 and HIF-1a expression in patients with HCC

**Figure 6:**
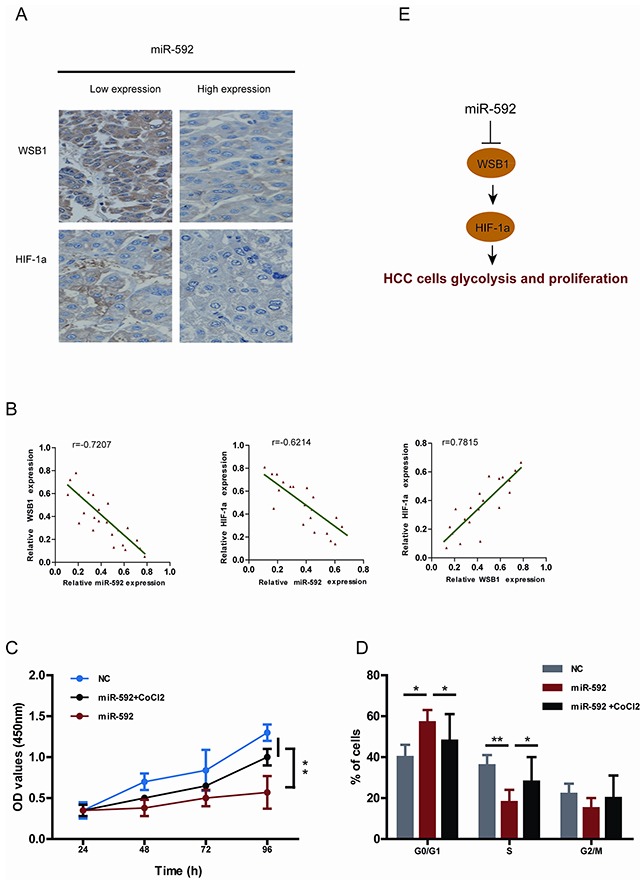
Clinical relevance of miR-592, WSB1 and HIF-1a expression in patients with HCC **A.** Representative images of IHC staining for WSB1 and HIF-1a in HCC patients with high and low miR-592 expression. **B.** Correlationship between miR-592 expression and WSB1 and HIF-1a expression in HCC patients tissues. The effect of miR-592 and CoCl2 on **C.** SK-Hep-1 cell proliferation and **D.** cell cycle progression. **E.** Schematic diagram of the regulatory pathway from miR-592/WSB1 / HIF-1a axis to glycolysis in HCC. The mean and SD of three independent experiments performed in triplicate are shown.

To further validate the inverse relationship between WSB1, HIF-1α and miR-592 in HCC cells, we examined miR-592, WSB1 and HIF-1α levels in the same human HCC tissues. As shown in Figure 6A, levels of both WSB1 and HIF-1α in human HCC tissues with high miR-592 expression were lower than those in HCC with low miR-592 expression. Consistently, we observed an inverse expression pattern between miR-592 and WSB1 or HIF-1α, whereas a positive expression pattern between WSB1 and HIF-1a was observed (Figure 6B). To further investigate whether the tumor suppressor role of miR-592 on HCC cells proliferation is mediated through disrupting the enhanced stability of HIF-1α, SK-Hep-1 cells were co-transfected with miR-592 mimics and treated with or without CoCl_2_. As shown in Figure 6C and Figure 6D, the inhibitory role of miR-592 in proliferation and cell cycle progression was reverted under the condition of treatment with CoCl_2_. Together, these results suggest that WSB1/HIF-1α axis is functional downstream of miR-592 in HCC. These data further support mechanism postulating that miR-592 knockdown directly promotes the expression of WSB1, thereby enhancing HIF-1α protein expression to maintain increased glycolysis in HCC (Figure 6E).

## DISCUSSION

miR-592 was recently proposed to be a new prognosis predictor for HCC patients and a new prospective target for HCC [14, 15]. However, the molecular mechanisms by which miR-592 exerts its role in tumor are not frequently studied. Herein, we report that the expression of miR-592 in HCC cells and tissues is downregulated, and miR-592 directly targets WSB1 mRNA. Our data suggest that decreased WSB1/HIF-1α expression via overexpression of miR-592 may decrease proliferation, glycolysis, and lactate production in HCC cells, which provides a new approach for HCC therapeutics.

The new finding of this study is that downregulation of miR-592 may facilitate HCC pathogenesis through the WSB1/HIF-1α axis. To uncover the molecular mechanism of miR-592 HCC inhibitory potential, we hypothesized that decreased WSB1 expression by miR-592 would reduce the HIF-1α level in HCC because HIF-1α signaling has been demonstrated to be enhanced by WSB1 in various cancers [10]. Indeed, we observed that the miR-592 knockdown-mediated upregulation of the WSB1 level increased the expression of HIF-1α, which plays an important role in critical aspects of HCC cancer biology, especially glucose metabolism [16, 17]. For example, its downstream signaling proteins GLUT1, HK1 and HK2 were upregulated to promote the glycolysis and pathogenesis of HCC, which was demonstrated by our and other reports [18]. Therefore, the newly identified miR-592/WSB1/HIF-1α axis provides new insight into the pathogenesis of HCC, suggesting a novel, potential therapeutic target for the treatment of HCC. The significance of this study is also underscored by the fact that miR-592 is frequently decreased in human HCCs, and that miR-592 knockout significantly promoted mouse HCC development. In future studies, it will be interesting to determine whether this newly discovered miR-592/WSB1/HIF-1α axis is distinct in HCC etiology.In addition, Given that CoCl_2_ only partially rescued miR-592 overexpression- caused cell proliferation retardation, and so did WSB-1 overexpression, this suggests that there may be alternative targets of miR592 that contributed to the growth retardation effect of miR-592.

Another important aspect of the current study is the novel role of miR-592 in inhibition of glycolytic phenotypes. Increased glycolysis has been shown being essential in promoting HCC progression. A recent report indicated that HCC patients with higher glucose uptake, had shorter survival times [19]. The expression of glycolytic genes, such as hexokinase (HK), PKM2, STAT3, HIF-1α, LDHA, Myc, and Glut1 were increased in HCC, and contributed to HCC growth [18]. Constantly, HCC with higher proliferation rate were accompanied with elevated expression of glycolytic genes [20]. However, very little was known about therapeutically targeting HCC metabolism, and more attention should be given to explore its essential role in HCC development.

Consistent with our data, a number of miRNAs have been recently shown to increase or inhibit tumor glucose metabolism. For example, through Kruppel-like factor 5, miR-133 was demonstrated to decrease the expression of GLUT4 in cardiomyocytes [21]. Similarly, in adipose tissues, miR-93 also directly associated with the 3′-UTR of GLUT4 and decreased its expression [22]. In head and neck carcinoma, miR-138 and miR-143 inhibited HK1 and HK2, respectively [23]. On the contrary, miR-290 cluster promotes glycolysis via upregulating Myc [24]. These findings suggest that targeting key metabolic enzymes is important for cancer therapy [25]. However, because targeting metabolic enzymes would have unacceptable effects on normal cells, such an anticancer strategy has also raised concerns [26]. In the present study, we showed that miR-592, by suppressing WSB1, but not key glycolytic genes, Myc, HK1 HIF-1α, HK2, stat3, or PKM2, effectively inhibits both glucose metabolism in HCC cells and the growth of xenografted HCC tumors, indicating a strong rationale for developing miR-592 as a novel metabolism-targeting therapeutic agent against HCC. In addition, two recent studies indicating that miR-592 inhibits prostate cancer and colorectal cancer growth [27, 28], further support miR-592 as a strongly general target candidate for anticancer therapies. Given that many of the HCC patients are often being excluded from chemotherapy or surgical resection due to their poor liver functions, thus, therapeutic against miR-592 may selectively provide a novel approach. Definitely, this is worth of further investigation – by improving the liver function and inhibiting tumor metabolisms, to benefit our HCC patients.

This is the first report that WSB1 promotes glycolysis and HCC cell proliferation through stabilization and activation of HIF-1a. And so far there is no report about the role of miRNAs in the expression of WSB1. Given that HIF-1α has been shown to up-regulate WSB1 in HCC combined with the evidence of WSB1 stabilizing HIF-1α [8, 9], there could be a positive feedback loop self-perpetuating the signaling between HIF-1α and WSB1. Because miR-529 presents as the sole upstream regulator of WSB1, it will be possible that mir-592 is a potential key regulator keeping the equilibrium of WSB1/HIF-1α signaling. Our finding also leads to two open questions: while the activation and stabilization of HIF isozymes has long been considered to promote tumorigenesis, what selectively drives HIF-1α activation and stabilization by WSB1 in HCC needs to be investigated further. Does HIF-1α as a transcription factor increase or decrease the expression of miR-592 in HCC?

In conclusion, the present study demonstrated a novel inhibitory role of miR-592 in HCC glycolysis by targeting WSB1/HIF-1α axis. Therefore, blocking this specific pathway may provide a novel approach for HCC therapeutics.

## MATERIALS AND METHODS

### Real-time quantitative reverse-transcription polymerase chain reaction

To measure the expression levels of mature miR-592, real-time quantitative RT-PCR was performed. The miRNAs were extracted from cultured cells or frozen tissues with a mirVana miRNA Isolation Kit (Applied Biosystems). Total RNA concentration was assessed by measuring absorbance at 260 nm using a NanoDrop spectrophotometer (ND-1000, Thermo Scientific). Using an Applied Biosystems 7500 Fast System and a TaqMan MicroRNA Reverse Transcription Kit (Applied Biosystems), ten nanograms of the extracted miRNAs were reverse-transcribed to cDNA. The reaction mixture was used for real-time RT-PCR of miR-592. U6 or GAPDH was used as an internal control. The primer sequences for miR-592 were 5′-CCATGACATTGTGTCAATATGCGA-3′ (Forward) and 5′-CGTCATGATGTTGCGTCACC-3′ (Reverse). For internal control U6 small nuclear RNA, (Forward) 5′-AGAGCCTGTGGTGTCCG-3′ and (Reverse) 5′-CAT CTTCAAAGCACTTCCCT-3′; (Forward) 5′-CATCACC ATCTTCCAGGAGCG-3′ and (Reverse) 5′-TGACCTT GCCCACAGCCTTG-3′ for GAPDH. HIF-1α: (Forward) 5′-ATTATTCTAGAACCGATTCACCATGGAGGGC-3′; (Reverse) 5′-TAAGAGGATCCTCAGTTAACTTGATCCAAAGCTCTGAG-3′. The reactive condition included preliminary denaturation at 95°C for 10 min, followed by 39 cycles of denaturation at 95°C for 15 s, annealing at 60°C for 1 min, followed by a final elongation step at 60°C for 10 min. Fold changes for the expression levels of miR-592 were calculated using the comparative cycle threshold method (2^−ΔCT^).

### Mouse models

Stably transfected cells (1.5 × 10^6^ in 0.2 mL) transfected with anti-miR-592 or negative control were injected subcutaneously into the flank region of 6 week-old male severe combined immunodeficiency mice (SCID; Institute of Laboratory Animal Sciences, Chinese Academy of Medical Sciences). Tumor growth was monitored every 5 days and tumors were measured with fine digital calipers and tumor volume was calculated by the following formula: tumor volume = 0.5 × width^2^ × length. After mice were killed, tumors were removed and weighed. The orthotopic HCC mouse model was established using indicated HCC cells. Briefly, SK-Hep-1 cells were transfected with a luciferase reporter plasmid. SK-Hep-1-luciferase cells were harvested from subconfluent cultures and resuspended in PBS. Suspensions consisting of single cells only, with >90% viability, were used for the injections. Animals were anaesthetised with 2.5% isoflurane/air mixture and injected intraperitoneally with 40 mg/mL D-luciferin potassium salt dissolved in PBS, according to the manufacturer's protocol. Then the animals were placed in the machine laterally, and a digital image was taken through the acquisition of pseudocolour images, which stand for the dimensional distribution of detectable photons from active luciferase generated spontaneously by the animals. All animal procedures were performed in accordance with protocols approved by the Institute Research Ethics Committee at University.

### Cell cycle analysis

Briefly, 24 h after transfection, cells were harvested and fixed in 70% ethanol and stored at −20°C for 6 h. Cells were then washed twice with ice-cold PBS and incubated with RNase A and PI (Sigma) for 30 min in the dark at room temperature. Cell cycle assays were performed using a flow cytometer (BD FACSCalibur, BD Bioscience). Cell cycle phase analysis was performed using the Modifit cell cycle analysis software (Becton Dickinson).

### Tissue specimens and cell lines

Paired HCC tissues and adjacent non-tumor tissues were collected from the Xijing Hospital of the Fourth Military Medical University. All tissue samples were flash-frozen in liquid nitrogen immediately after collection and stored at −80°C until use. Both tumor and non-tumor samples were confirmed by pathological examination. The study was approved by the Ethics Committee of the Xijing Hospital of the Fourth Military Medical University, and informed consent was obtained from each patient according to the committee's regulations. Human HCC cell lines were purchased from American Type Culture Collection (ATCC), cultured in Dulbecco's modified Eagle's medium (Gibco) supplemented with 10% fetal bovine serum, and maintained at 37°C in a humidified incubator containing 5% CO_2_.

### Cell transfection

The miR-592 inhibitors (Cat. HSTUD0817, Sigma), miR-592 mimics (Cat. HMI0817, Sigma) and nonspecific nucleotide sequences as a negative control (NC, Sigma) were used. For transfection, 1 × 10^5^ HCC cells were cultured into 6-well plate in growth medium without serum and antibiotics, and incubated overnight, then transfected with indicated oligonucleotides using HiPerFect Transfection Reagent (Qiagen) according to the manufacturer's protocol. The mixture was added to cells at a final concentration of 100 nM. After incubation for 4–6 h, the serum-free medium was removed, and cells were maintained in DMEM (Gibco) containing 10% FBS.

### Western blotting and antibodies

Cells or tissues were washed with phosphate buffer solution and solubilized in lysis buffer. Protein concentration was determined by the Bio-Rad Protein Assay kit (Bio-Rad). Cell lysate was separated on a 8% or 10% SDS-PAGE and transferred to polyvinylidene difluoride membranes (Millipore). Then, nonspecific binding was blocked with 5% non-fat milk for 1 h. The membrane was incubated with indicated antibody followed by appropriate secondary antibodies, and visualized by enhanced chemiluminescence (ECL, Amersham Pharmacia Biotech). The Mouse polyclonal anti-WSB1 antibody (ab68953) was purchased from Abcam. The rabbit polyclonal anti-HIF-1α, GAPDH (sc-367714), Glut1 (sc-377228), HK2 (sc-130358) antibodies were purchased from Santa Cruz.

### Cellular glucose-6-phosphate and ATP production

The cellular levels of glucose-6-phosphate was measured using a Glucose-6-phosphate Fluorometric Assay kit (Cayman). Intracellular ATP levels were measured using ATP Bioluminescence Assay Kit (Roche Applied Science). Briefly, 5 × 10^5^ cells were lysed with boiling lysis reagent and supernatant was collected. Fifty microliters of diluted sample were mixed with 50 μL of luciferin/luciferase reagents. Luminescence was measured using Luminoskan Ascent (Thermo Scientific).

### Determination of glucose consumption and lactate production

Cells were cultured in DMEM without phenol red for 15 h, and the culture media was then harvested for measurement of lactate or glucose concentrations. Lactate levels were quantified using the Lactate Assay kit (BioVision) and glucose levels were determined with using a glucose assay kit (Sigma-Aldrich). All values were normalized to total protein levels (BCA Protein Assay Kit, Thermo Scientific).

### miRNA target prediction

The bioinformatic analysis of miRNA predicted targets was determined by using three different algorithms: PITA and miRanda.

### Immunohistochemistry

In brief, tissue sections were dewaxed in xylene and rehydrated through graded ethanols. Then the sections quenched in 3% methanolic peroxide for about 10 min to block endogenous peroxidase activity. Immunohistochemical localization of indicated proteins was carried out using commercially available specific antibodies (cell signalling technology). Staining was visualized using the Biovision IHC kit according to the manufacturer's suggested protocol. The slides were counterstained with hematoxylin (Sigma–Aldrich).

### Cell proliferation assay

Indicated HCC cells (2 × 10^4^) were seeded in 96-well plates. Then, 10 μL CCK8 (Cell Counting Kit-8: CK04-13, Dojindo Molecular Technologies, Inc.) solution was added to each well, and incubated at 37°C for 4 h. Mitochondrial dehydrogenase within the cells reduced CCK8 solution to a yellow product called formazan. The amount of formazan produced in the reaction sample is positively correlated with cell viability. And the optical density (OD) values were measured at 450 nm using a scanning multi-well spectrophotometer (Bio Rad Model 550).

### Luciferase reporter assay

For luciferase reporter experiments, a fragment containing part of WSB1 3′-UTR was amplified by PCR from human cDNA using primers: forward 5′-TTAATTAAACGACACAAAACTACTAC-3′ and reverse 5′-GTAGTAGTTTTGTGTCGTTTAATTAA-3′. This fragment was inserted into the pGL3 control vector with a TK promoter immediately downstream from the luciferase stop codon by *Xba*I/*Sal*I digestion generating the pGL3/WSB1-3′UTR reporter plasmid. The WSB1 3′-UTR seed sequence was mutated using the QuickChange II Site-directed mutagenesis kit (Stratagene) according to the manufacturer's instructions. Changes introduced by mutagenesis were confirmed by DNA sequencing. Indicated cells were transfected with the pGL3/WSB1-3′UTR reporter plasmid or the empty construct using Lipofectamine LTX reagent (Invitrogen) according to the manufacturer's protocol. Briefly, transient transfections were performed in subconfluent cells seeded in six-well plates using 1 μg/well of the luciferase reporter plasmid, 0.03 μg/well of pRL-CMV (Promega) as an internal control, and 3 μl of Lipofectamine LTX. The activities of firefly and Renilla luciferases were determined in cell lysates using the Dual-Luciferase^®^ Reporter Assay System (Promega) and a luminometer (Orion I, Berthold Detection Systems) according to the manufacturer. Results were expressed as the ratio of firefly to renilla activity.

### Statistical analysis

All statistical analyses were conducted using SPSS 19.0 software (SPSS Inc.). Data are expressed as mean ± SD (unless otherwise stated). Significant differences were established by Student's t-test or one-way ANOVA, according to the number of groups compared, using the GraphPad Instat program (GraphPad Software V2.03, GraphPad Software Inc.). In the latter case, when significant variations were found, the Tukey–Kramer multiple comparison test was applied. ^*^P < 0.05, ^**^P < 0.01, ^***^P < 0.001.

## SUPPLEMENTARY FIGURES AND TABLE




