# Supplementary material for: miR-592/WSB1/HIF-1α axis inhibits glycolytic metabolism to decrease hepatocellular carcinoma growth
Source: Oncotarget. 2016 May 2;7(23):35257–69. doi: 10.18632/oncotarget.9135 (PMC5085226; doi:10.18632/oncotarget.9135)
Supplement: Supplementary file 1 [file oncotarget-07-35257-s001.pdf]

miR-592/WSB1/HIF-1α axis inhibits glycolytic metabolism to decrease hepatocellular carcinoma growth

SUPPLEMENTARY FIGURES AND TABLE

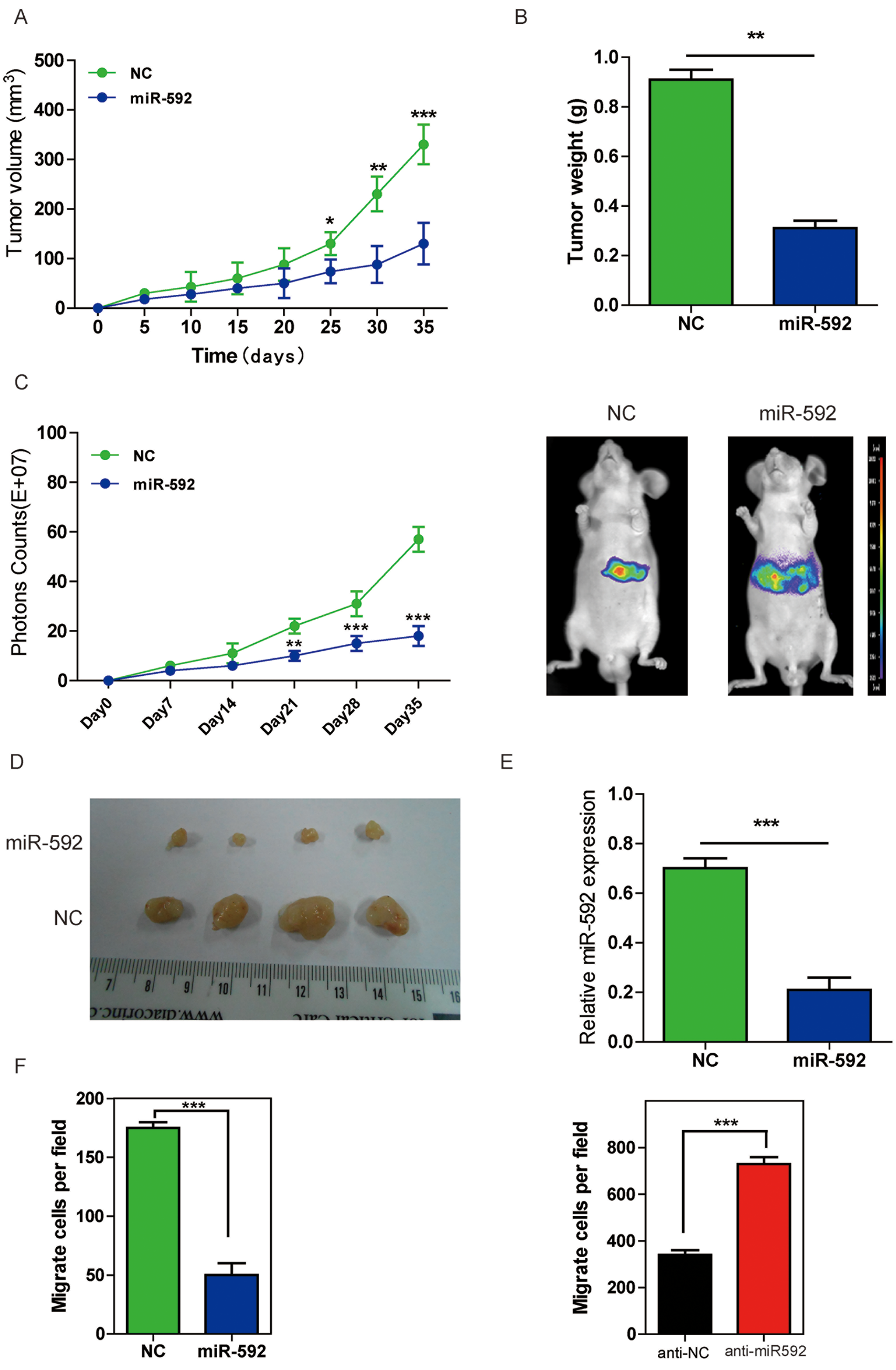

Supplementary Figure S1:

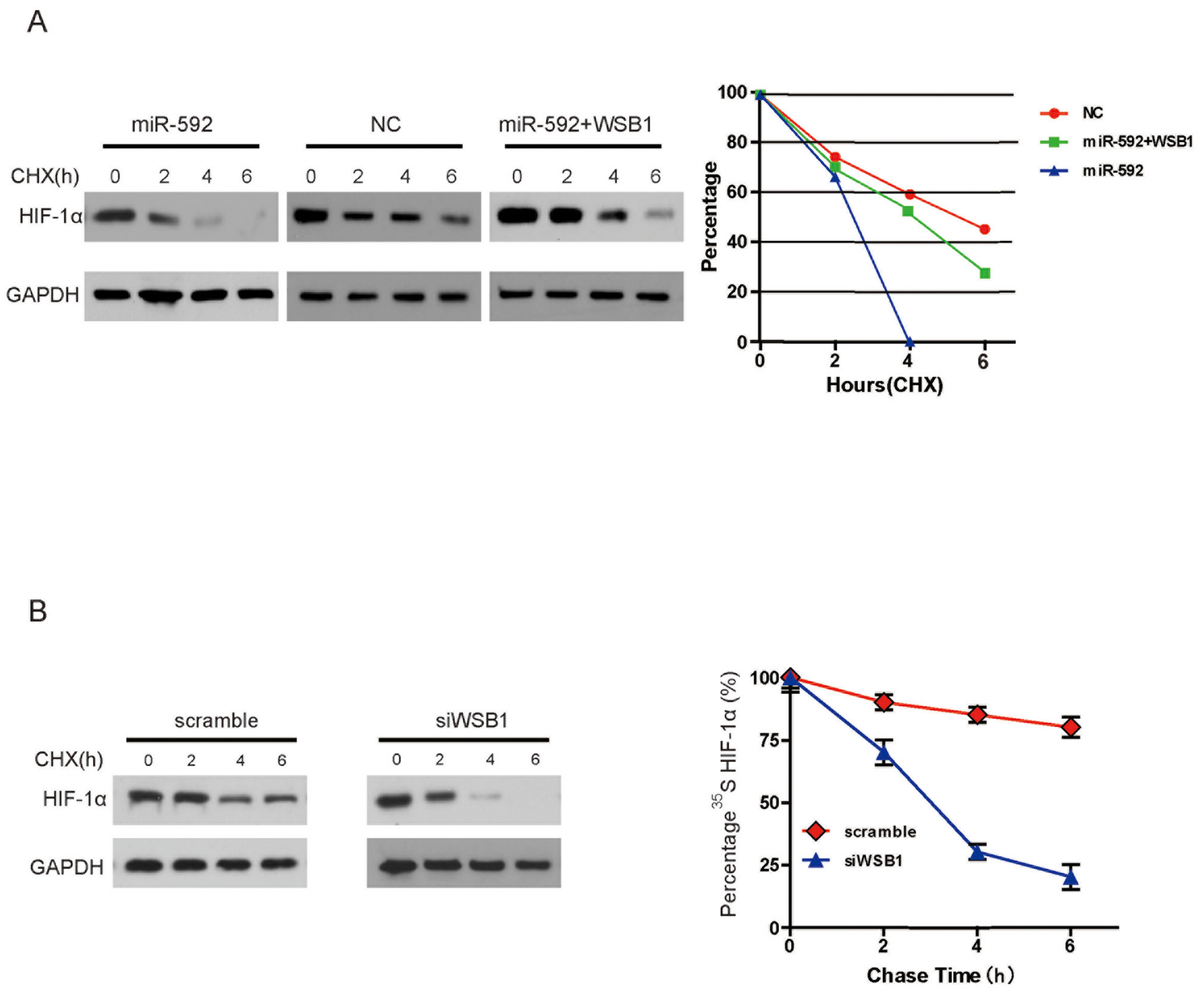

Supplementary Figure S2:

Supplementary Table S1:

See Supplementary File 1
